# COMPARISON OF SENSOR BASED CONTINUOUS ADL ASSESSMENTS TO CARE-GIVER ADCS-ADL SURVEY RESULTS

**DOI:** 10.1093/geroni/igad104.2808

**Published:** 2023-12-21

**Authors:** John Fitch, Rachel Williams, Dennis Myers, Michael Devin

**Affiliations:** Birkeland Current, Waco, Texas, United States; Birkeland Current, Waco, Texas, United States; Baylor University, Waco, Texas, United States; Birkeland Current, Waco, Texas, United States

## Abstract

This paper presents analysis results between the Birkeland Current (BC) Sovrin IoT in-home sensing system and monthly ADCS-ADL survey data for Care Recipient/Caregiver (CR/CG) pairs (N=117) as part of a Phase II NIA SBIR study (R44AG065118) conducted from 2021 to 2023. The analysis provides correlation analysis between overall BC ADL Scores and ADCS-ADL scores as well as comparisons to IADL/BADL scores. The CR population was screened to represent mild to mid-level cognitive decline and was continuously monitored in home and assisted living environments for up to 18 months. The BC system demonstrates significant correlation with the ADCS-ADL score at the level of sensitivity of the ADCS survey and subfactor results. The BC system is able to demonstrate sensitivity to a single point change in the two-factor BADL score as well as a single point change in the two-factor IADL score. Discussion of limitations of comparison to retroactive surveys is described. Novel ADL measurement dimensions are discussed for further study.

